# Supplementary material for: Associations between sleep duration and depression, mental health, physical health, and general health in U.S. adults: A population-based study
Source: PLoS One. 2026 Jan 14;21(1):e0321347. doi: 10.1371/journal.pone.0321347 (PMC12803467; doi:10.1371/journal.pone.0321347)
Supplement: S1 File — Contains Supplementary Figure 1 (Trends in Sleep Duration Categories), Supplementary Figure 2 (Prevalence of Depression by Sleep Duration), and Supplementary Tables 1–5 describing baseline characteristics, treatment effects, mentally and physically unhealthy days, and predicted general health probabilities. (DOCX) [file pone.0321347.s001.docx]

**SUPPLEMENTARY FILES**

**Supplementary Figure 1**


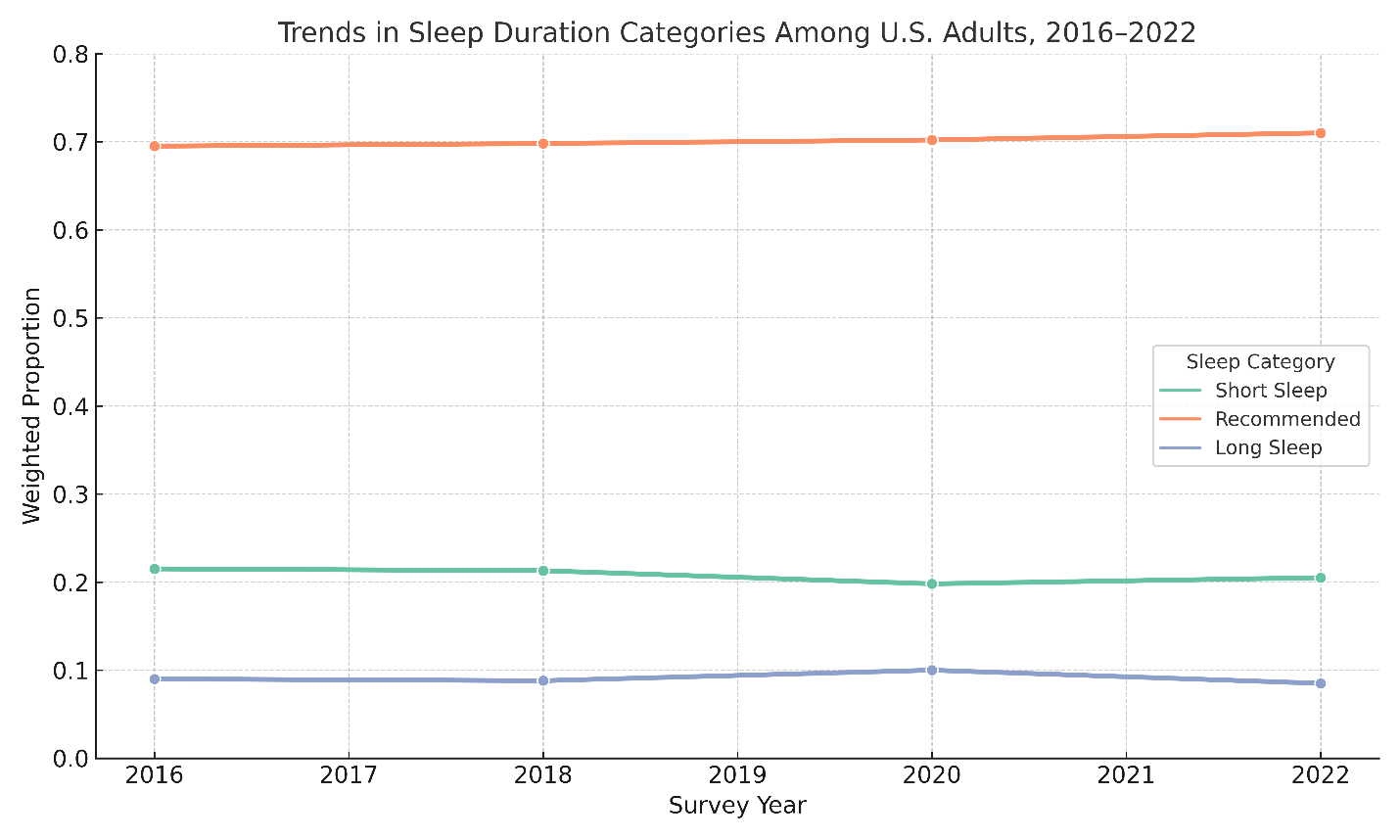


**Supplementary Figure 1. Trends in Sleep Duration Categories Among U.S. Adults, 2016–2022**

This figure displays the weighted prevalence of self-reported sleep duration categories among U.S. adults using data from the Behavioral Risk Factor Surveillance System (BRFSS) for survey years 2016, 2018, 2020, and 2022. Sleep duration was classified as: Short Sleep (≤5 hours), Recommended Sleep (6–8 hours), and Long Sleep (≥9 hours). Recommended sleep steadily increased from 69.5% in 2016 to 71.0% in 2022, while short sleep showed a modest decline over time. Long sleep remained relatively stable. Weighted estimates reflect national sampling weights and are restricted to survey years in which the complete sleep module was administered. Shaded bands represent 95% confidence intervals.

**Supplementary Figure 2. Estimated Prevalence of Depression by Sleep Duration Category**

**
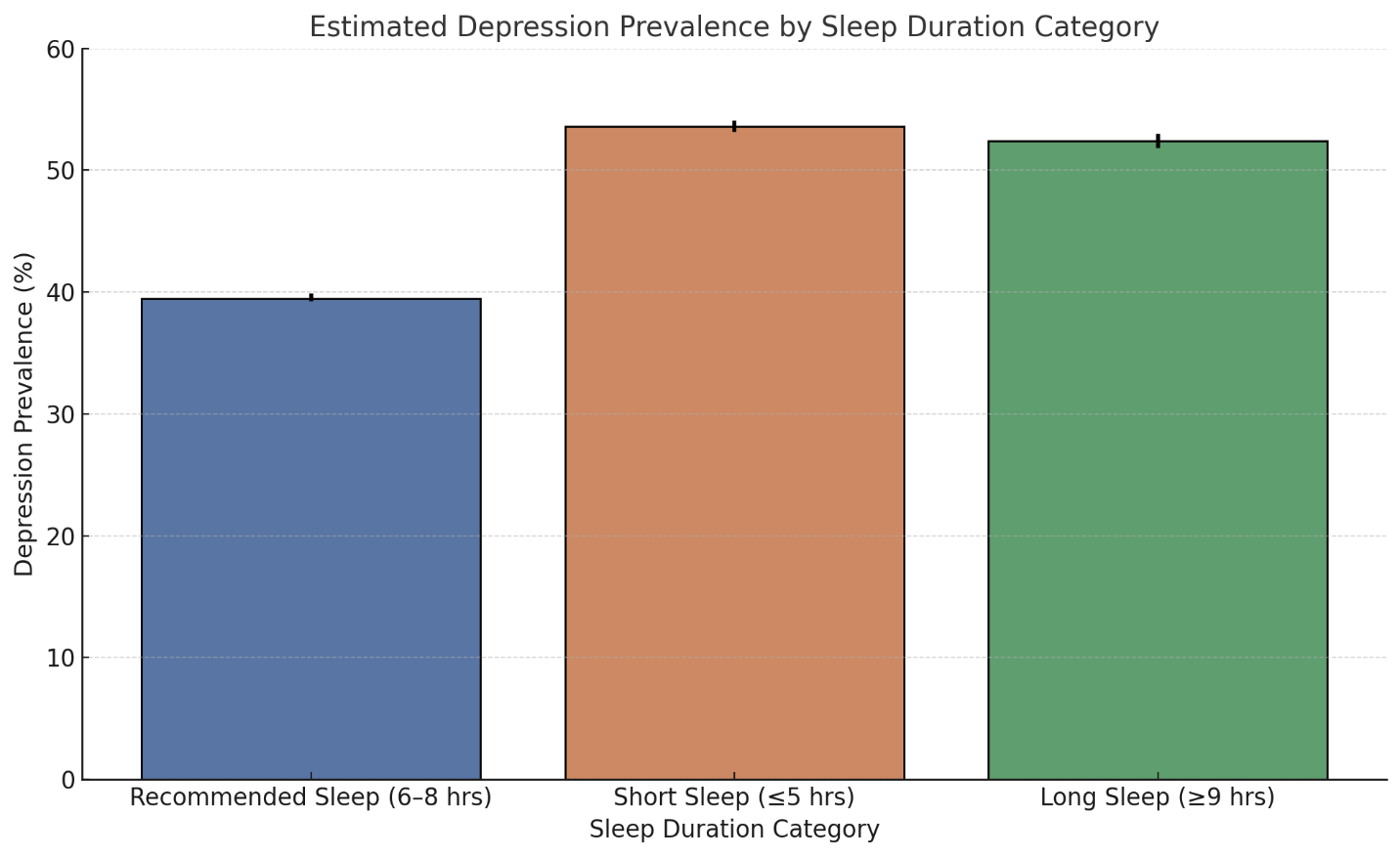
**

This figure shows the estimated prevalence of self-reported depression among U.S. adults based on sleep duration, using inverse probability weighting (IPW) models. Individuals who sleep the recommended 6–8 hours per night serve as the reference group with a baseline depression prevalence of 39.5%. Those with short sleep (≤5 hours) had an estimated prevalence of 53.6%, while those with long sleep (≥9 hours) had a prevalence of 52.4%. Error bars represent 95% confidence intervals. Models were adjusted for age, sex, race/ethnicity, BMI, education, income, marital status, state, and survey year.

**Supplementary Table 1 — Baseline Characteristics by Sleep Duration**

Supplementary Table 1 summarizes demographic and health characteristics of 318,886 adults according to sleep duration category. Recommended sleepers (7–9 hours) comprised the largest group (49.7%), followed by short sleepers (≤6 hours; 44.6%) and long sleepers (≥10 hours; 5.7%). Long sleepers were most likely to be older (65.2% ≥55 years) and previously married (41.7%), whereas recommended sleepers were more commonly college graduates (40.9%) and had higher household income (>100K: 39.0%). Short sleepers showed a higher prevalence of obesity (43.1%) and lower income. Racial composition also differed significantly, with White adults more frequently reporting recommended sleep (80.5%). Mental and physical health indicators showed gradient patterns across groups: mean mentally unhealthy days were highest among long sleepers (16.3 days) and lowest among recommended sleepers (10.4 days). Similarly, mean physically unhealthy days were greatest in the long sleep group (17.6 days). Depression was most prevalent among long sleepers (61.7%), followed by short sleepers (52.4%) and recommended sleepers (41.7%). Reported general health also varied, with poor health highest among long sleepers (30.6%). All comparisons were statistically significant (p<0.001).

Supplementary Table 1: Sociodemographic and Health Characteristics of the Study Population by Sleep Duration Category (BRFSS 2016-2023)

| **Variables (N=318,886)** | **Total Population** | **Short Sleep** | **Recommended Sleep** | **Long Sleep** | **p-value** |
| --- | --- | --- | --- | --- | --- |
|  | (N=318,886) | (n=142,313) (44.6%) | (n=158,557) (49.7%) | (n=18,016) (5.7%) |  |
| **Age** |  |  |  |  | <0.001 |
| 18-34Yr. | 66, 151 (20.7%) | 27,916 (19.6%) | 36,047 (22.7%) | 2,188 (12.4%) |  |
| 35-54Yr. | 101,844 (31.9 %) | 51,336 (36.1%) | 46,432 (29.3%) | 4,076 (22.6%) |  |
| >55Yr. | 150,891 (47.3%) | 63,061 (44.3%) | 76,078 (48.0%) | 11,752 (65.2%) |  |
| **Sex** |  |  |  |  | <0.001 |
| Male | 117,044 (36.7%) | 53,936 (37.9%) | 56,758 (35.8%) | 6,350 (35.3%) |  |
| Female | 201,610 (63.3%) | 88,265 (62.1%) | 101,707 (64.2%) | 11,638 (64.7%) |  |
| **BMI** |  |  |  |  | <0.001 |
| Underweight | 6,558 (2.2%) | 3,037 (2.3 %) | 3,042 (2.1%) | 479 (2.9%) |  |
| Normal | 82,030 (28.0%) | 32,712 (25.0%) | 45,157 (30.9%) | 4,161 (25.1%) |  |
| Overweight | 89,225 (30.4%) | 38,712(29.6%) | 45,991 (31.5%) | 4,522 (27.3%) |  |
| Obese | 115,661 (39.4%) | 56,299(43.1 %) | 51,964 (35.6 %) | 7,398 (44.7 %) |  |
| **Race/Ethnicity** |  |  |  |  | <0.001 |
| White | 241,731 (76.5%) | 101,668 (72.2%) | 126,537 (80.5 %) | 13,526 (75.8%) |  |
| Black | 27,350 (8.7%) | 15,320 (10.9%) | 10,073 (6.4%) | 1,957 (11.0 %) |  |
| Hispanic | 18,936 (6.0%) | 9,29 (6.6%) | 8,799 (5.6%) | 842 (4.7 %) |  |
| Other | 27,856 (8.8%) | 14,493 (10.3%) | 11,848 (7.5%) | 1,515 (8.5%) |  |
| **Education** |  |  |  |  | <0.001 |
| High School or less | 113,071 (35.6%) | 56,419 (39.8%) | 48,274 (30.5%) | 8,378 (46.7%) |  |
| Some college | 96,360 (30.3%) | 45,642 (32.2%) | 45,234 (28.6%) | 5,484 (30.5%) |  |
| College graduate | 108,587 (34.1%) | 39,816 (28.1 %) | 64,674 (40.9 %) | 4,097 (22.8 %) |  |
| **Household Income ($)** |  |  |  |  | <0.001 |
| <25K | 50,917 (19.0%) | 27,475 (29.9%) | 18,999 (14.3 %) | 4,443 (30.2%) |  |
| 25-50K | 62,187 (23.2%) | 30,464 (25.4%) | 27,416 (20.6 %) | 4,307 (29.3%) |  |
| 50-100K | 67,421 (25.2%) | 29,198 (24.4%) | 34,841(26.1%) | 3,382 (23.0%) |  |
| >100K | 87,182 (32.6%) | 32,645 (27.3%) | 51,976 (39.0%) | 2,561(17.4%) |  |
| **Marital Status** |  |  |  |  | <0.001 |
| Married | 149,465 (47.2%) | 62,011 (43.9%) | 80,542 (51.1%) | 6,912 (38.7%) |  |
| Previously Married | 97,385 (30.8%) | 47,938 (33.9%) | 41,985 (26.7%) | 7,462 (41.7%) |  |
| Never Married | 69,751 (22.0%) | 31,276 (22.1%) | 34,965 (22.2%) | 3,510 (19.6%) |  |
| **Covid Era** |  |  |  |  | <0.001 |
| pre-Covid | 167,209 (52.4%) | 75,857(53.3%) | 81,529 (51.4%) | 9,823 (54.5%) |  |
| post-Covid | 151,677 (47.6%) | 66,456 (46.7%) | 77,028 (48.6%) | 8,193 (45.5%) |  |
| **Outcomes** |  |  |  |  |  |
| Depression | 150,152 (47.6%) | 73,697 (52.4%) | 65,483 (41.7%) | 10,972 (61.7%) | <0.001 |
| Poor Mental Health days | 12.7 + 10.7 | 14.9 + 11.0 | 10.4+ 9.9 | 16.3 + 11.1 | <0.001 |
| Poor Physical Health days | 12.3 + 11.2 | 13.9 + 11.4 | 10.3+ 10.5 | 17.6 + 11.3 | <0.001 |
| **General Health** |  |  |  |  | <0.001 |
| Excellent | 17,331 (5.5%) | 5,493 (3.9%) | 11,361 (7.2%) | 477 (2.7%) |  |
| Very Good | 67,436 (21.2%) | 22,940 (16.2%) | 42,856 (27.1%) | 1,640 (9.1%) |  |
| Good | 100,540 (31.6%) | 43,095 (30.4%) | 53,326 (33.7%) | 4,119 (22.9%) |  |
| Fair | 86,257 (27.1%) | 44,285 (31.2%) | 35,742 (22.3%) | 6,230 (34.7%) |  |
| Poor | 46,614 (14.7%) | 26,161 (18.4%) | 14,962 (9.5%) | 5,491 (30.6%) |  |

**Supplementary Table 2 — Association Between Sleep Duration and Depression**

Supplementary Table 2 presents the average treatment effects of sleep duration on depression. After adjusting for demographic and socioeconomic variables, both short and long sleep were associated with significantly elevated probability of depression compared with recommended sleep. Short sleep was linked to an 8.9-percentage-point higher predicted probability of depression (ATE = 0.089; 95% CI, 0.081–0.097), whereas long sleep demonstrated a substantially larger excess probability of 19.6 percentage points (ATE = 0.196; 95% CI, 0.176–0.217). Recommended sleepers had a predicted depression probability of 0.405 (95% CI, 0.399–0.410). These findings indicate that both insufficient and prolonged sleep are strongly associated with higher depression burden, with long sleep showing the greatest adverse effect.

## Supplementary Table 2. Average Treatment Effects of Sleep Duration on Depression.

| Outcome | Sleep Categories | Coef. | Std. Err. | z-value | 95% CI |
| --- | --- | --- | --- | --- | --- |
| Depression | Short Sleep vs Recommended | 0.089 | 0.004 | 21.93 | 0.081–0.097 |
|  | Long Sleep vs Recommended | 0.196 | 0.010 | 19.00 | 0.176–0.217 |
|  | Recommended Sleep | 0.405 | 0.003 | 145.60 | 0.399–0.410 |

*This table reports the estimated Average Treatment Effects (ATE) of sleep duration categories on the probability of depression, using inverse probability weighting (IPW) models. The reference group is individuals with recommended sleep duration (7–9 hours per night). Coefficients represent the estimated mean difference in predicted probability of depression compared to the recommended sleep group. The model was adjusted for demographic and socioeconomic variables, including age, sex, race/ethnicity, BMI, education, income, marital status, geographic region, and survey year. Reported values include the coefficient (Coef.), standard error (Std. Err.), z-value, and 95% confidence interval (CI).*

**Supplementary Table 3 — Association Between Sleep Duration and Mentally Unhealthy Days**

Supplementary Table 3 shows that short and long sleep are associated with clinically meaningful increases in mentally unhealthy days. Relative to recommended sleepers (11.18 days), short sleepers reported an additional 3.49 mentally unhealthy days in the past month (ATE = 3.493; 95% CI, 3.329–3.657), while long sleepers reported 5.10 extra days (ATE = 5.101; 95% CI, 4.656–5.546). Thus, long sleep was associated with the largest increase in psychological symptom burden. These patterns are consistent with a graded worsening in mental well-being as sleep duration deviates from recommended levels.

## Supplementary Table 3. Average Treatment Effects of Sleep Duration on Mentally Unhealthy Days.

| Outcome | Sleep Categories | Coef. | Std. Err. | z-value | 95% CI |
| --- | --- | --- | --- | --- | --- |
| Mental Health Days | Short Sleep vs Recommended | 3.493 | 0.084 | 41.76 | 3.329–3.657 |
|  | Long Sleep vs Recommended | 5.101 | 0.227 | 22.47 | 4.656–5.546 |
|  | Recommended Sleep | 11.177 | 0.059 | 187.99 | 11.060–11.293 |

*This table reports the estimated Average Treatment Effects (ATE) of sleep duration categories on the number of self-reported mentally unhealthy days in the past 30 days, using inverse probability weighting (IPW) models. The reference group is individuals with recommended sleep duration (7–9 hours per night). Positive coefficients indicate a greater number of mentally unhealthy days relative to the recommended sleep group. The model adjusts for age, sex, race/ethnicity, BMI, education, income, marital status, geographic region, and survey year. Reported values include the coefficient (Coef.), standard error (Std. Err.), z-value, and 95% confidence interval (CI).*

**Supplementary Table 4 — Association Between Sleep Duration and Physically Unhealthy Days**

Supplementary Table 4 evaluates the relationship between sleep duration and physically unhealthy days. Compared with recommended sleep (10.08 days), short sleep was associated with 2.25 additional physically unhealthy days per month (ATE = 2.245; 95% CI, 2.086–2.403), whereas long sleep was associated with 5.44 additional days (ATE = 5.444; 95% CI, 5.014–5.873). Thus, long sleep duration exhibited the strongest association with physical symptom burden. These findings suggest that deviations from recommended sleep duration are linked to worse physical health, with particularly marked increases among long sleepers.

## Supplementary Table 4. Average Treatment Effects of Sleep Duration on Physically Unhealthy Days.

| Outcome | Sleep Categories | Coef. | Std. Err. | z-value | 95% CI |
| --- | --- | --- | --- | --- | --- |
| Physical Health Days | Short Sleep vs Recommended | 2.245 | 0.081 | 27.76 | 2.086–2.403 |
|  | Long Sleep vs Recommended | 5.444 | 0.219 | 24.83 | 5.014–5.873 |
|  | Recommended Sleep (POmean) | 10.078 | 0.059 | 170.24 | 9.962–10.194 |

*This table reports the estimated Average Treatment Effects (ATE) of sleep duration categories on the number of self-reported physically unhealthy days in the past 30 days, using inverse probability weighting (IPW) models. The reference group is individuals with recommended sleep duration (7–9 hours per night). Coefficients represent the estimated mean difference in physically unhealthy days compared to the recommended sleep group. Positive coefficients indicate a greater number of poor physical health days relative to recommended sleepers. The model adjusts for demographic and socioeconomic variables, including age, sex, race/ethnicity, BMI, education, income, marital status, geographic region, and survey year. Reported values include the coefficient (Coef.), standard error (Std. Err.), z-value, and 95% confidence interval (CI).*

**Supplementary Table 5 — Predicted Probabilities of General Health Status by Sleep Duration**

Supplementary Table 5 presents adjusted predicted probabilities of general health outcomes. Recommended sleepers demonstrated the most favorable health profile, with the highest probabilities of reporting excellent (0.077) and very good health (0.258). In contrast, both short and long sleepers exhibited lower probabilities of excellent health (0.049 and 0.033, respectively) and higher predicted probabilities of poor health (0.149 and 0.203, respectively). Long sleepers demonstrated the largest burden, with the highest prevalence across fair and poor health categories. The probability of good health was modestly higher among recommended sleepers (0.335) compared with short (0.325) and long (0.300) sleepers. Overall, these results confirm a U-shaped association, where both short and long sleep durations are linked to poorer self-rated health relative to recommended sleep.

# Supplementary Table 5: Adjusted Predicted Probabilities of General Health Categories by Sleep Duration

| GENHLTH | Sleep Category | Predicted Probability | Std. Err. | z-value | 95% CI |
| --- | --- | --- | --- | --- | --- |
| Excellent | Recommended | 0.077 | 0.001 | 64.41 | 0.075–0.080 |
| Excellent | Short | 0.049 | 0.001 | 53.48 | 0.047–0.051 |
| Excellent | Long | 0.033 | 0.001 | 26.68 | 0.031–0.036 |
| Very Good | Recommended | 0.258 | 0.002 | 132.92 | 0.254–0.261 |
| Very Good | Short | 0.197 | 0.002 | 112.67 | 0.194–0.201 |
| Very Good | Long | 0.151 | 0.004 | 40.17 | 0.143–0.158 |
| Good | Recommended | 0.335 | 0.002 | 176.22 | 0.331–0.338 |
| Good | Short | 0.325 | 0.002 | 177.88 | 0.321–0.328 |
| Good | Long | 0.300 | 0.003 | 99.75 | 0.294–0.306 |
| Fair | Recommended | 0.231 | 0.002 | 136.64 | 0.227–0.234 |
| Fair | Short | 0.280 | 0.002 | 145.79 | 0.276–0.284 |
| Fair | Long | 0.313 | 0.003 | 102.38 | 0.307–0.319 |
| Poor | Recommended | 0.100 | 0.001 | 79.22 | 0.097–0.102 |
| Poor | Short | 0.149 | 0.002 | 95.67 | 0.146–0.152 |
| Poor | Long | 0.203 | 0.005 | 40.52 | 0.194–0.213 |

*Table presents adjusted predicted probabilities for each general health category by sleep duration derived from IPW models.*
