# Supplementary material for: Associations between sleep duration and depression, mental health, physical health, and general health in U.S. adults: A population-based study
Source: PLoS One. 2026 Jan 14;21(1):e0321347. doi: 10.1371/journal.pone.0321347 (PMC12803467; doi:10.1371/journal.pone.0321347)
Supplement: Attachment — Submitted filename: Response to Reviewers_Sleep7232025.docx [file pone.0321347.s004.docx]

We sincerely thank the reviewers for their thoughtful comments, detailed reviews, and valuable suggestions. We have implemented all the requested revisions and have outlined them below. In addition, we made a few other changes that we believe further enhance the clarity, relevance, and overall quality of the manuscript**.**

**Summary of Major Revisions:**

1. **New Analytical Approach:**
   We revised the analytic design from a binary classification of sleep duration (short vs. recommended) to a **three-category classification**:
   - Short Sleep (≤5 hours),
   - Recommended Sleep (6–8 hours), and
   - Long Sleep (≥9 hours).
     This allows for a more nuanced evaluation of the association between sleep and health outcomes.
2. **Expanded Health Outcomes (Now Four):**
   We now include **General Health Status** (fair/poor vs. good/very good/excellent) as a fourth outcome in addition to:
   - Depression,
   - Poor mental health days, and
   - Poor physical health days.
3. **Revised Sample Size and Flow Diagram:**
   The analytic sample is now **N = 318,000**. A detailed **study flow diagram** (Figure 1) has been added to illustrate inclusion/exclusion criteria and missing data handling.
4. **Precise Variable Definitions:**
   We now clearly provide the **exact BRFSS questions** and operational definitions used to derive each variable and outcome, as requested.
5. **Revised Manuscript Title:**
   The title has been updated to:
   ***“Associations Between Sleep Duration and Depression, Mental Health, Physical Health, and General Health in U.S. Adults: A Population-Based Study”***
   to better reflect the revised scope and objectives.

**Point-by-Point Responses**

**Editorial Comments**

**1. Citation Formatting**

*“Please ensure that all citations adhere to the formatting style specified in the journal’s author guidelines especially in-text citations.”*

**Response:**
All references have been reformatted in accordance with PLOS ONE citation guidelines. In-text citations now use the correct numerical format.

**2. Temporal Trend Justification**

*“The analysis does not include any temporal stratification or trend assessment... A visual or statistical analysis of trends would significantly enhance the contribution of the study.”*

**Response:**
We agree with this recommendation and have now included a temporal trend analysis to examine the changes in sleep duration and health outcomes across 2016–2023. A new section has been added to the Methods and Results, and the table is available in Table 2 and Supplementary Figure .

**3. Propensity Score Matching (IPW) Methodology Description**

*“The methodology is not adequately described… include rationale, variables, matching technique, and diagnostics.”*

**Response:**
We have added a new subsection to the Methods section titled “Propensity Score Matching (Inverse Probability Weighting – IPW)”. This subsection outlines the rationale for using IPW to reduce selection bias inherent in observational data. We detail the covariates included in the propensity score model (age, sex, race, education, BMI, income, etc.), the matching algorithm used (1:1 nearest neighbor without caliper), and the approach for assessing covariate balance (standardized mean differences). In addition, we have provided the full Stata syntax used for implementing the IPW procedure to enhance transparency and reproducibility.

**4. Sample Size Discrepancy (Table 1 vs. Text)**

*“Clarify how the N = 163,724 was derived and ensure internal consistency.”*

**Response:**
We have clarified that Table 1 originally reflected the matched sample. It has now been updated to explicitly indicate whether the characteristics pertain to the matched or unmatched population, and we have included the full analytic sample size of 318,000. Additionally, a study flowchart has been added to illustrate the sample derivation and exclusion criteria. A comprehensive description of all study variables, stratified by sleep category, is now provided. As we employed inverse probability weighting (IPW) rather than traditional propensity score matching, we did not construct separate matched and control groups; instead, the entire weighted sample was used for analysis.

**5. Add Line Numbers**

*“Include line numbers.”*

**Response:**
Line numbers have been included in both the clean and tracked-changes versions of the revised manuscript.

**Reviewer #2 Comments**

**Comment 1: COVID-19 Bias Potential**

*“The period in which data was collected includes the COVID pandemic. Can the authors address how their findings were impacted…?”*

**Response:**
We conducted a stratified analysis comparing the pre-pandemic (2016–2019) and pandemic (2020–2023) periods to assess potential variation in associations. Results remained consistent across both timeframes. Additionally, we included year and state fixed effects in our models to account for temporal and geographic variability. A binary variable for the COVID-19 pandemic period (“COVID”) was also created and included in the models to control for the specific effects of the pandemic years. These adjustments are detailed in the revised Methodology and Results section.

**Comment 2: Benchmark Outcomes with National Survey (e.g., Udupa et al. 2023)**

*“Add more info regarding the prevalence of outcomes compared to other surveys…”*

**Response:**
We have expanded the Discussion section to include comparisons of our findings with Udupa et al. (2023) and other national BRFSS-based studies. Our estimates are consistent with prior literature, reinforcing external validity.

**Comment 3: Impact of Covariates**

*“Add the impact of covariates (sex, age, etc.) in the models – can be done in supplementary.”*

**Response:**
We appreciate the reviewer’s insightful suggestion. While we agree that examining the independent effects of covariates such as sex, age, education, income, and race on each health outcome would be valuable, we have chosen not to include these detailed estimates in the current manuscript for several reasons.

First, incorporating these results across all four outcome variables would significantly expand the number of tables (we currently present six tables), potentially overwhelming readers and detracting from the study’s main focus—examining associations between sleep duration and health outcomes.

Second, the purpose of this study was to evaluate sleep duration as the primary exposure of interest, while adjusting for key covariates. Including full covariate-specific estimates may shift focus away from our central objective.

That said, we fully acknowledge the importance of these covariate effects and have adjusted for all suggested variables in our multivariable models. To address this important line of inquiry, we are actively planning a separate follow-up study that will comprehensively evaluate the independent impact of sociodemographic covariates on depression, mental health, physical health, and general health status using the BRFSS dataset.

We hope this rationale is acceptable and appreciate your understanding.

**Comment 4: Clarify Use of ‘Poor Physical Health Days’ vs. Physical Activity**

*“The survey asks about physical activity, yet you report poor physical health days.”*

**Response:**
We clarified that our outcome was **BRFSS-reported number of poor physical health days**, not physical inactivity. The specific question wording is now quoted in the Methods section.

**Comment 5: Add Exact Question Wording & Abstract Sample Size**

*“Please add the exact phrasing used for sleep duration… and participant number in the abstract.”*

**Response:**
We have added the precise BRFSS item:

*“On average, how many hours of sleep do you get in a 24-hour period?”*
to the Methods section, and the final sample size (N = 318,000) is now included in the Abstract.

**Comment 6: Add Visualizations Beyond Tables**

*“Can the authors find a way to visualize the results?”*

**Response:**
We have included the following visualizations to support our findings and enhance interpretability:

- **Supplementary Figure 1**: A line graph illustrating the weighted proportion of each sleep duration category (Short, Recommended, Long) among U.S. adults across BRFSS survey years 2016–2023.
- **Supplementary Figure 2**: A bar chart displaying the estimated prevalence of self-reported depression across sleep categories, derived from inverse probability weighting (IPW) models.

These figures highlight both temporal trends in sleep patterns and the adjusted associations between sleep duration and depression.

- A **flow diagram** showing the analytic sample derivation (Figure 1).

**Additional ScreenIT & Editorial Compliance Updates**

- **Study Flow Chart:** Added (Supplementary Figure 1).
- **Inclusion/Exclusion Criteria:** Individuals were excluded if they had missing data on sleep duration, any study outcomes, or covariates. Additionally, participants under the age of 18 were excluded from the analysis.
- **Sex as a Variable:** Included in descriptive stats and multivariable models.
- **Open Data:** Minimal dataset will be provided upon acceptance. De-identified BRFSS data and codebook are publicly available.
- **ORCID ID for Corresponding Author:** Updated in Editorial Manager profile.

We hope the comprehensive revisions and additions have addressed all comments satisfactorily. We are grateful for the opportunity to revise and improve our work and look forward to your favorable consideration.

Sincerely,
**Oluwasegun Akinyemi, MD, MSc. PhD**
Senior Research Fellow
Howard University College of Medicine
Oluwasegun.akinyemi@howard.edu
(309) 255-8284
